# Gut Microbes Reveal *Pseudomonas* Medicates Ingestion Preference *via* Protein Utilization and Cellular Homeostasis Under Feed Domestication in Freshwater Drum, *Aplodinotus grunniens*

**DOI:** 10.3389/fmicb.2022.861705

**Published:** 2022-05-26

**Authors:** Changyou Song, Haibo Wen, Guangxiang Liu, Xueyan Ma, Guohua Lv, Ningyuan Wu, Jianxiang Chen, Miaomiao Xue, Hongxia Li, Pao Xu

**Affiliations:** ^1^Key Laboratory of Freshwater Fisheries and Germplasm Resources Utilization, Ministry of Agriculture and Rural Affairs, Freshwater Fisheries Research Center, Chinese Academy of Fishery Sciences, Wuxi, China; ^2^Wuxi Fisheries College, Nanjing Agricultural University, Wuxi, China

**Keywords:** feed domestication, microbe, *Pseudomonas*, protein utilization, *Aplodinotus grunniens*

## Abstract

With strong demand for aquatic products, as well as a rapid decrease in global fishery resources and capture fisheries, domesticating animals to provide more high-quality proteins is meaningful for humans. Freshwater drum (*Aplodinotus grunniens*) is widely distributed in the wild habitats of North America. However, the research on *A. grunniens* and the feed domestication with diets composed of artificial compounds remains unclear. In this study, a 4-month feeding domestication experiment was conducted with *A. grunniens* larvae to evaluate the underlying mechanism and molecular targets responsible for alternations in the ingestion performance. The results indicated that a significant increase in the final body weight was exhibited by the feed domesticated group (DOM, 114.8 g) when compared to the group that did not ingest the feed (WT, 5.3 g) as the latest version we raised From the result, the final body weight exhibited significant increase between unfavorable with the feed (WT, 5.3 g) and feed domesticated group (DOM, 114.8 g). In addition, the enzyme activity of digestive enzymes like amylase, lipase, and trypsin was increased in DOM. Genes related to appetite and perception, such as *NPY4R*, *PYY*, and *LEPR*, were activated in DOM. 16s rRNA gene sequencing analysis revealed that *Pseudomonas* sp. increased from 58.74% to 89.77% in DOM, which accounts for the dominant upregulated microbial community at the genus level, followed by *Plesiomonas*. Analogously, *Mycobacterium*, *Methylocystis*, and *Romboutsia* also accounted for the down-regulated microbes in the diversity. Transcriptome and RT-PCR analysis revealed that feed domestication significantly improved protein digestion and absorption, inhibited apoptosis by AGE-RAGE signaling, and activated extracellular matrix remodeling by relaxin signaling. Integrated analysis of the microbiome and host transcriptome revealed that *Pseudomonas-*mediated ingestion capacity, protein utilization, and cellular homeostasis might be the underlying mechanism under feed domestication. These results indicate *Pseudomonas* and its key genes relating to food ingestion and digestion could serve as the molecular targets for feed domestication and sustainable development in *A. grunniens*.

## Highlights

- DOM improves appetite and perception.
- Protein metabolism and cellular homeostasis were active to DOM.
- *Pseudomonas* was increased under DOM.
- *Pseudomonas* medicates ingestion and growth under DOM.

## Introduction

With the fast-growing population and rising prosperity all over the world, producing sufficient amounts of animal-based food products has become a critical challenge for humankind (FAO, 2020). In accordance with livestock animal products, aquatic animals contribute significantly to meet the demand of protein requirements. Therein, aquaculture is, globally, the fastest growing farmed food production sector, showing a 5.3% increase annually from 2001 to 2018 (FAO, 2020). However, there are still some bottlenecks in the development of aquaculture in recent years. One of the most important limitations is that the majority of aquaculture production is from a small number of culture species (Du et al., 2021), as over 90% of the production involves just 27 species/species groups (Hallerman et al., 2021). Therefore, domestication of some species with excellent biological, nutritional, social, and economic traits will promisingly promote sustainable aquaculture development.

During the domestication of new aquatic species, feed utilization or ingestion performance is the first and vital step (Liao and Huang, 2000). Domestication with artificial feed is a process that involves adaptation to the ingestion habits, which is closely related to feeding motivation, metabolic and physiological change, neuroendocrine system regulation, and behavioral adaptations (Volkoff, 2016). The gut system is closely related to nutrient absorption and has been confirmed to impact the ingestion behavior and host appetite adaptation (Dabrowski and Portella, 2005). Therefore, to improve the heritable adaption to ingestion habits from the aspects of the gut system is the key question to answer for feed domestication.

In recent years, microorganisms have been increasingly recognized for their role in the health and overall performance of aquatic organisms (Parata et al., 2021). Gut microbiota refers to the entire population of microorganisms that colonize the intestine, and investigations on microbiota associated with a fish gut have deepened our knowledge about the complex interactions that occur between microbes and host fish (Talwar et al., 2018). Functionally, gut microbiota exerts a profound influence on immunity, nutrient-processing capacity, disease susceptibility, growth, and reproduction of the host animals (Nayak, 2010; Ghanbari et al., 2015). Inversely, the gut microbial community is also affected by the nutritional status (Xia et al., 2014), diet situations (Peredo et al., 2015), and feeding behaviors (Ye et al., 2014). Therefore, alternation of gut microbiota is vital to uncover the ingestion behavior under domestication.

Discovering the core microbiome is the primary goal for many researchers interested in understanding gut microbial communities (Turnbaugh et al., 2007), which is critical for their application to promote the host health and wellbeing by manipulating the microbes inhabiting the aquatic animals. However, defining a core microbiome has proven to be an elusive task in many species, including humans, and terrestrial and aquatic animals. Therefore, the diversity and abundance of dominated microbes are of great importance for the evaluation of the functions of gut microbiome. However, the studies on the role of gut microbiota in ingestion performance and appetite adaption are currently limited.

Freshwater drum (*Aplodinotus grunniens*) is a kind of fish that is endemic to North and Central America. It is the only species in the genus of *Aplodinotus* that exclusively inhabits freshwater for its entire life (Hernández-Gómez et al., 2021). With respect to edibility, the freshwater drum is featured to possess a higher edible proportion, with delicious and nutritious flesh rich in proteins, amino acids, and fatty acids. Moreover, the freshwater drum has no intermuscular bones, which improves the fish quality and processing of the aquatic product. Therefore, these distinct characteristics reveal freshwater drum has the potential for domestication and cultivation to provide high-quality proteins for human beings. However, the domestication, management practices, aquaculture, and even scientific research for this particular species is limited. With these prospects, we imported the larvae of freshwater drum from the United States in 2016, and have achieved a milestone in the artificial breeding technique and cultivation in 2019, which provided a break through and prospects for aquaculture. In behavior, freshwater drum is similar to carnivorous fish, and the diet for freshwater drums in the wild is generally benthic animals and composed of macroinvertebrates (mainly larvae of aquatic insects and bivalve mussels), as well as small fish, mollusks, and crayfish in certain ecosystems (Rypel, 2007; Jacquemin et al., 2014). In our cultivation, we found freshwater drum prefers live baits, and the ingestion ability and performance of artificial diets that contain high levels of plant-source proteins are limited. However, economically, the feeding behavior and food preference determine the production costs and profitability of freshwater drum aquaculture. Therefore, the domestication of a freshwater drum by an artificial diet enriched with plant-source proteins is critical for the sustainable development of the fish, particularly to expand the scale of breeding, aquaculture, and food processing.

In the present study, we evaluated the relationship between the gut microbiome and ingestion performance under feed domestication in the freshwater drum. Meanwhile, the regulation between gut microbes and encoding genes was also investigated. These results could reveal the feed ingestion regulation and could provide potential targets to improve the feed domestication and sustainable development of freshwater drums.

## Materials and Methods

### Ethics Statement

This study was approved by the Animal Care and Use Committee of Nanjing Agricultural University (Nanjing, China). All animal procedures were performed according to the Guidelines for the Care and Use of Laboratory Animals in China.

### Experimental Animals and Feed Domestication

The embryos of *A. grunniens* were obtained from the Freshwater Fisheries Research Center, Chinese Academy of Fishery Sciences. Larvae were raised in tanks at 25 ± 2°C and fed with chironomid larvae (*Chironomus tentans*). After 21 days of hatching, about 60,000 healthy larvae were randomly transferred into three outdoor fish ponds (pond size was 667 m^2^, 20,000 individuals per pond, representing three biological replicates) for feed domestication experiment. During the experiment, fish in the WT and DOM groups were fed with commercial diets (45% crude protein and 8% crude lipid; Seahorse feed Ltd., China) four times a day. The domestication experiment was conducted for 4 months (16th May to 15th September), and the water source was drawn from underground. Aeration was also provided to maintain enough dissolved oxygen (DO). The water quality was maintained as follows: pH 7.6–7.8, DO > 6 mg/L, NH_3_ < 0.01 mg/L, and H_2_S < 0.01 mg/L.

### Sample Collection

After 4 months of feed domestication experiment, fish were starved for 24 h to evacuate the residual feed in the alimentary tract prior to sampling. The fish that ingested the compounds of the feed exhibited increased size, while the non-ingested fish were obviously small. Therefore, 27 non-feed ingested (WT) and feed ingested (DOM) fish in each pond were randomly taken to evaluate the growth performance indices, respectively. Meanwhile, nine WT and DOM fish were randomly taken and anesthetized with tricaine mesylate (MS-222, 100 mg/L) for sample collection, respectively. Blood samples were obtained from the caudal vein and stored in tubes coated with heparin, and then centrifuged at 4,500 rpm at 4°C for 10 min to obtain the plasma. Meanwhile, the sampled fish were dissected on ice to collect the intestinal tissue. The whole intestine and intestinal contents were collected and immediately frozen in liquid nitrogen and stored at −80°C for mRNA sequencing, 16s sequencing, and RT-PCR analysis.

### Growth Performance and Evaluation of Digestive Enzyme Activity

Growth performance was evaluated by measuring final body weight (FBW), condition factor (CF), viscerosomatic index (VSI), and hepatosomatic index (HSI). Each parameter was calculated as follows: CF = Final body weight/Final body length^3^; VSI (%) = 100 × Viscera weight/Final body weight; HSI (%) = 100 × Liver weight/Final body weight.

The glucose content in the plasma and the activity of digestive enzymes, such as amylase, lipase, and trypsin, in the intestine were determined by using assay kits according to the manufacturer’s protocol (provided by Nanjing Jiancheng Bioengineering Institute, China). Briefly, glucose was detected by hexokinase method (Category No: F006-1-1), amylase was determined by starch-iodine colorimetry method (Category No: C016-1-1), lipase was determined by colorimetric method (Category No: A054-1-1), and trypsin was determined by ultraviolet colorimetry method (Category No: A080-2-2).

### RNA Extraction and Transcriptome Sequencing

Nine samples of whole-intestine tissues from WT and DOM groups were selected to conduct the high-throughput sequencing analysis. Then, three fish intestine samples from each group were randomly mixed, and three biological replicates were finally applied to extract the total RNA according to the established methods described by Song et al. (2017).

#### cDNA Library Construction and *de novo* Sequencing

The library construction and sequencing were conducted according to our previously established methods (Song et al., 2021). Briefly, total RNA was subjected to quality control with Ailent 2100 and Nanodrop (ThermoFisher Ltd.), and samples with 1.8 < OD260/280 < 2.0, 28S/18S > 1.0, and RNA integrity number (RIN) > 1.8 were treated with oligo (dT) to enrich mRNA. Next, random primers were used to splice the mRNA into short fragments (200–700 nt). Short fragments were used as templates to synthesize cDNA, and PCR amplification was performed after adding “A” tail and sequencing connector. Finally, paired-end sequencing strategies were applied for *de novo* high-throughput sequencing with Illumina Hiseq™ 2000 platform.

#### Raw Data Dominate, *de novo* Assembly, and Annotation

After sequencing, adaptors and low-quality reads were removed by using FastQC software (version: 0.10.8) to get the clean reads. Clean reads were assembled with Trinity, and the transcripts were mapped to NR, Swiss Port, Pfam, KOG, and GO databases for annotation.

#### Enrichment of Differentially Expressed Genes

Read Count and FPKM (fragments per kilobase million) for each annotated transcript were calculated using RSEM (version: 1.3.1), and transcripts with | Fold Change| ≥ 2 and corrected *P*-value < 0.05 were recognized as the differentially expressed genes (DEGs). Meanwhile, DEGs were also subjected to gene ontology (GO) and KEGG enrichment analysis using Blast2GO.

### Gut Microbe Identification

#### Microbial DNA Extraction and 16S Sequencing

The intestinal tissues and contents from three fishes were randomly pooled as a replicate, and five replicates in each group were selected for gut microbiome analysis. Microbial DNA from each replicate was extracted by using E.Z.N.A.^®^ Soil DNA Kit (Omega Bio-Tek, Norcross, GA, United States) according to the manufacturer’s protocol (Sun et al., 2020). The integrity of isolated DNA was measured by Nanodrop ND2000 spectrophotometer (Thermo Scientific, United States) and 1% agarose gel electrophoresis. Meanwhile, the concentration of isolated DNA was measured with Quant-iT PicoGreen dsDNA Assay Kit (Invitrogen, United States) and fluorometer, and diluted to 20 ng/μl for sequencing.

The full length of 16S rRNA was amplified using the primers 338 F (5′-ACTCCTACGGGAGGCAGCAG-3′) and 806 R (5′-GGACTACHVGGGTWTCTAAT-3′) by PCR and sequenced in PacBio Sequel System using SMRT cell protocol. Sequences were denoised using DADA2 (version 1.8) and assembled into amplicon sequence variants (ASVs). A representative sequence of each ASV was assigned to a taxonomic level in the Ribosomal Database Project (RDP) database using the RDP classifier. Principal component analysis and heatmap analysis were performed by using the R package (version 3.1.0).

#### 16s Sequencing Data Analysis

The alpha diversity was calculated using QIIME software (version 1.9.1), which included Ace, Chao, Shannon, and Simpson. One-way analysis of variance (ANOVA) was performed for all diversity indices, followed by a Tukey’s *post hoc* test when statistically significant differences were observed (*P* < 0.05) using SPSS 25.0. The enriched chord diagram and PCoA diagram were drawn using the R package (version 3.1.0). A genera abundance table was loaded into Primer v5, and a similarity percentage (SIMPER) analysis was performed to determine the genera responsible for differences between the groups. The cut-off for low contributions was set to a default of 90%. We then used SourceTracker2 (Knights et al., 2011), a Bayesian community-level microbial source-tracking tool, to estimate the proportion of sequences in different groups (Liu et al., 2021).

### Integrated Analysis Between Differentially Expressed Genes and Microbes

To explore the relationship between differential DEGs and microbes, the functional prediction of differential OTUs and DEGs was integrated. With Pearson analysis, the same or similar pathways that were shared between the two sets of data were selected, and the obtained pathways were regarded as DEG-related functions that were affected by the intestinal microbiota.

### RT-PCR Analysis

Total RNA was extracted from nine whole-intestine or brain tissues in each group using RNAiso Plus reagent (Dalian Takara Co., Ltd., China), and was incubated with RNase-free DNase (Dalian Takara Co., Ltd., China) to remove the contaminating genomic DNA. Quantity and quality of the RNA were assessed by OD260/280 method and electrophoresis in 1.5% agarose gel. Primers (listed in Table 1) for each gene were designed using Primer Premier 5.0 based on the mRNA sequences obtained from *A. grunniens* intestine genome database in our lab. All primers were synthesized by Shanghai Generay Biotechnology, Co., Ltd., China. Real-time quantitative PCR (RT-PCR) was performed with the SYBR^®^ Primix Ex TaqTM II (TliRNase Plus) Kit using ABI 7500 Real-time PCR System according to the manufacturer’s protocol. The relative expression levels of the target genes were normalized to the housekeeping *A. grunniens* gene β-actin and further calculated using the 2^–ΔΔCT^ method.

**TABLE 1:** Primers and sequences referred in the experiments.

Gene	Primer	Sequence (5′→3′)	Amplification size (bp)	Gene	Primer	Sequence (5′→3′)	Amplification size (bp)
*NPY4R*	F	AGCACAACACCAACCACAAC	159	*FQN60*	F	GGAATCTCCACCGACTTTCA	248
	R	TGACAAACACGAGGCAAGAG			R	CACAACCCTCGATCCACTTC	
*GHR*	F	GTCCTGACCCACCAGTGTCT	174	*GNAO1*	F	GAGGAGAAGAAGGCGAGGAT	239
	R	CTTCCCAGTTTGTCGCATTT			R	CCGTGGAGGACAAACTTCAT	
*PYY*	F	ACATGCTGAGATCGTGGATG	242	*GNB5*	F	GTGTTGGGTGCTCTCTCCAT	245
	R	AGAATCACCACCGAACAGGA			R	TTGTTATCCAAGCCACCACA	
*LEPR*	F	GTGCGTTCGTTCAGTGTGTT	182	*PKCB*	F	GCAAGCAGAAGACCAAGACC	200
	R	CGGTCAGTGTAGGGCAGTTT			R	CCATCCATCTACGCCTTGTT	
*CREB1*	F	CATGCCCACTCCCATCTATC	181	*CD36*	F	GGAAATCCTGTGGGGTTACA	210
	R	GTCGCTGGTCTGAGCGTACT			R	GAAGCATCTGTCCCGTTGAT	
*FRA2*	F	CCCACAATCAATGCAATCAC	249	*COL6A1*	F	AGAGAATGGGGAGCAAGGTT	203
	R	CCTCACCCTCCTTTTCTCCT			R	TGATTCCTCTCGGTCCTTTG	
*C/EBPA*	F	CCTGTCCGGGTTTAGGTTCT	163	*ITGA6*	F	CTCACTCGCCCTTCAAACTC	182
	R	CAGGTCGATGGAAGTCTCGT			R	TCCACAGCAGAAACACAAGC	
*PP1B*	F	AACGTGGACAGCCTCATCTC	224	*ITGB1*	F	AAACATTCACCGAGCCTGAC	231
	R	GGGAAGCCACCATACTCAAA			R	TGTCCCATATTGCCAGCTTT	
*Bax*	F	GAGGTGGTGGAACATCTGCT	209	*LAMC1*	F	ACAGGAGGGTCAACGACAAC	157
	R	TTGGTGGTCAGTGCCTTGTA			R	GCCTTCATCTTGGCCTCTTT	
*PKCA*	F	GGAGTAACCACACGCACCTT	210	*C1QTNF9*	F	GGCATCTTCTCCTGTCCTGT	196
	R	CAGGGACTTTGGGTAGGACA			R	CCCAGACCTCATCGTGTTTT	
*PLCD1*	F	GACGAATGGGATCGAAAAGA	196	*MEP1A*	F	CGACTTCAGCAAGATGGACA	154
	R	ATCGAGGGAAAAGGTCTGGT			R	GCTCTTTGTGTGGACCCAGT	
*STAT3*	F	GCTCCTGAGTCTGAGATTGGA	164	*SLC15A1*	F	CTGAGTTTGCCGAGAAGGAG	153
	R	TTGGCTTCAGCTTCATTGTG			R	TCTTGGTCTGGCTGGTTTTC	
*TGFBR2*	F	AAGAGCAGGGCTTTGAGACA	183	*SCL3A1*	F	GACCCAGACAAACCTCCAGA	214
	R	TCGGCAGGTAGGCTGTAATC			R	CCTGTGGGGTGCCATAATAC	
*VEGFAA*	F	CAGGCCGATGTTTATTCCAT	163	*SLC7A9*	F	GTGGCTGGTAGAGAGGGTCA	177
	R	CGGGTCTTCCTCTGTCTCTG			R	CAGACCATAAAACGCCCACT	
*ATF4* × *1*	F	CTGGGGAGTGAAGTGGATGT	220	β*-actin*	F	CCTCTCTGTCCACCTTCCAG	165
	R	AGTCGCTTGATGGAGGAGAG			R	GTGGTGTGTGGTTGTTTTGC	
*c-FOS*	F	CACCATCTCCAACAGCACAG	154				
	R	CTCCCAGTCCTGAGTCGTGT					

*The mRNA sequences for each gene were obtained from A. grunniens intestine transcriptome sequencing database. Primers for RT-PCR were designed using Primer Premier 5.0.*

### Statistical Analysis

Independent samples *t*-test was conducted to analyze the transcriptional expression of related genes in SPSS 25.0. Meanwhile, all the data were validated for normality and homogeneity for variances. All results were expressed as the mean ± standard error of the mean (mean ± SEM).

## Results

### Feed Domestication Improves Growth Performance and Digestive Enzyme Activity in *A. grunniens*

**FIGURE 1:**
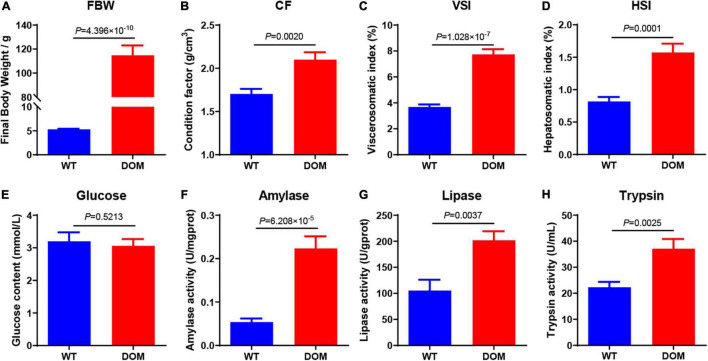
Feed domestication improves growth performance and digestive enzyme activity in *A. grunniens*. Panels **(A–D)** represent the growth performance, panel **(E)** represents the glucose content in the plasma, and panels **(F–H)** represent the digestive enzyme activity in the intestine. **(A)** Final body weight, FBW; **(B)** Condition factor, CF; **(C)** Viscerosomatic index, VSI; **(D)** Hepatosomatic index, HSI; **(E)** Glucose; **(F)** Amylase; **(G)** Lipase; and **(H)** Trypsin. Data were analyzed by Student’s *t*-test, and results were indicated as mean ± SEM; **(A–D)** *n* = 27; **(E–H)** *n* = 9.

The growth performance was first evaluated. We found the fish domesticated with compound feed (DOM) showed a significant improvement in the final body weight (FBW, Figure 1A, *P* = 4.395 × 10^–10^), increased by about 21.66-fold when compared to the fish that were unfavorable with the feed (WT). Meanwhile, the condition factor (CF, Figure 1B, *P* = 0.0020), viscerosomatic index (VSI, Figure 1C, *P* = 1.028 × 10^–7^), and hepatosomatic index (HSI, Figure 1D, *P* = 0.0001) were all significantly increased in the DOM group when compared to the WT group.

In accordance with the growth performance, digestibility and absorption property were also detected. Plasma glucose content exhibited no significant difference between the groups (Figure 1E, *P* = 0.5213). However, the activities of intestinal amylase (Figure 1F, *P* = 6.208 × 10^–5^), lipase (Figure 1G, *P* = 0.0037), and trypsin (Figure 1H, *P* = 0.0025) were significantly increased in the DOM group.

### Feed Domestication Improves Appetite and Perception in *A. grunniens*

**FIGURE 2:**
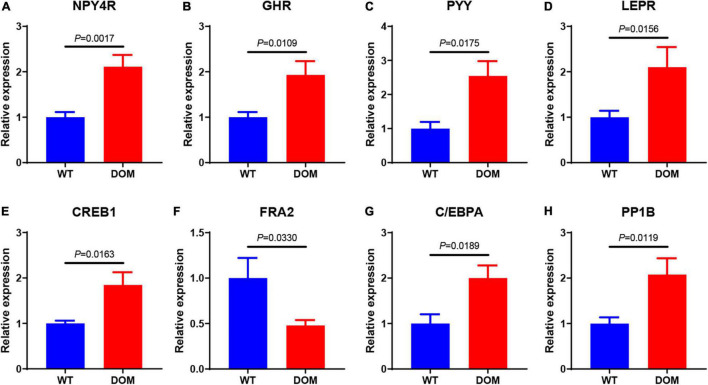
Feed domestication improves appetite and perception in the brain of *A. grunniens*. Transcriptional expression of appetite-related **(A–D)** and perception-related **(E–H)** genes. **(A)** NPY4R, **(B)** GHR, **(C)** PYY, **(D)** LEPR, **(E)** CREB1, **(F)** FRA2, **(G)** C/EBPA, and **(H)** PP1B. Data were analyzed by Student’s *t*-test, and results were indicated as mean ± SEM, *n* = 9.

To obtain an overview regarding the growth performance, digestive capacity, and food performance, we evaluated the transcriptional expression of appetite- and perception-related genes. Key genes related to appetite, including *NYP4R* (Figure 2A, *P* = 0.0017), *GHR* (Figure 2B, *P* = 0.0109), *PYY* (Figure 2C, *P* = 0.0175), and *LEPR* (Figure 2D, *P* = 0.0156), were all significantly upregulated in the intestine of DOM. The expression of learning- and memory-related genes that function to feed perception were also detected; the expression of *CREB1* (Figure 2E, *P* = 0.0163), *C/EBPA* (Figure 2F, *P* = 0.0330), and *PP1B* (Figure 2G, *P* = 0.0189) were upregulated, while that of *FRA2* (Figure 2H, *P* = 0.0119) was downregulated in the brain tissue of DOM.

### Transcriptomic Analysis of *A. grunniens* Intestine Domesticated With Compound Feed

**FIGURE 3:**
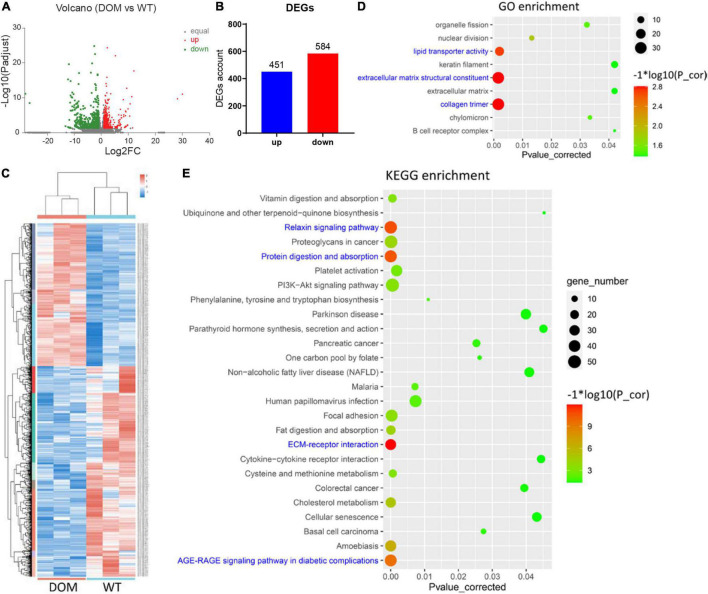
Transcriptomic analysis of *A. grunniens* intestine domesticated with compound feed. **(A)** Volcano plot of unigenes; **(B)** Statistics of differentially expressed genes (DEGs); **(C)** Heatmap cluster of DEGs; **(D)** GO annotation of DEGs; and **(E)** KEGG enrichment of DEGs.

To reveal the underlying mechanism of food performance, we performed *de novo* transcriptomic analysis with high-throughput sequencing. When compared to WT, a total of 1,035 differentially expressed genes (DEGs) were identified and annotated in DOM (| Fold Change| ≥ 2 and corrected *P*-value < 0.05), including 451 upregulated and 584 downregulated genes (Figures 3A–C and Supplementary Tables 1A,B). To uncover the molecular functions of these DEGs, GO and KEGG enrichment analyses were conducted. With the threshold of corrected *P*-value < 0.05, a total of 9 GO items (Figure 3D and Supplementary Table 2A) and 26 KEGG pathways (Figure 3E and Supplementary Table 2B) were enriched. Specifically, collagen trimer, structural constituent of extracellular matrix, and lipid transporter activity were the most significantly enriched GO items (corrected *P*-value < 0.01), indicating DOM altered the cytoskeleton and cellular lipid transport. In accordance with GO enrichment, ECM–receptor interaction, relaxin signaling pathway, protein digestion and absorption, and AGE-RAGE signaling pathway were the most significantly enriched KEGG pathways (corrected *P*-value < 1 × 10^–10^), revealing DOM affected protein utilization, extracellular structure, and cell fate determination.

### Microbial Composition of *A. grunniens* Intestine Domesticated With Compound Feed

**FIGURE 4:**
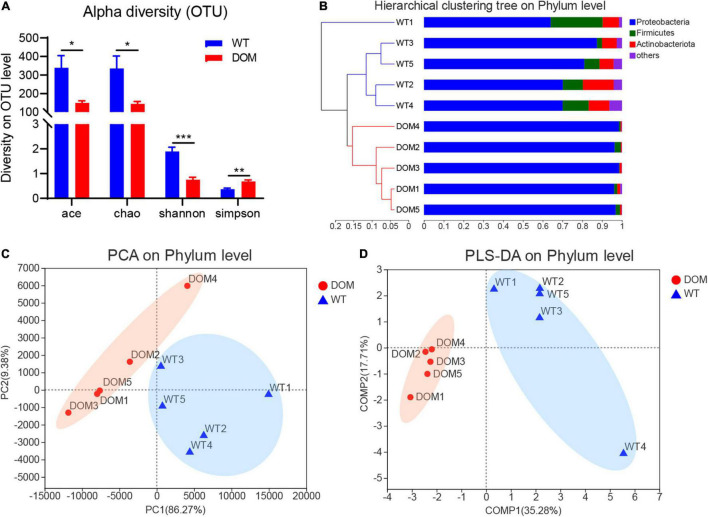
Microbial composition of *A. grunniens* intestine domesticated with compound feed. **(A)** Alpha density of the operational taxonomic units (OTUs); **(B)** Hierarchical cluster at phylum level; **(C)**, Principal coordinates analysis (PCA) at the phylum level; and **(D)** Partial least-squares discriminant analysis (PLS-DA) at the phylum level. * represent *P* < 0.05; ** represent *P* < 0.01; and *** represent *P* < 0.001.

To further explore the potential regulatory mechanism, 16s rRNA gene amplicon sequencing was used to determine the composition of the microbial community, and alpha and beta diversity indices present in the fish gut contents. A total of 943 operational taxonomic units (OTUs) were identified (Supplementary Table 3), corresponding to 26 phyla, 78 classes, 189 orders, 286 families, 458 genera, and 657 species. Ace, Chao, Shannon, and Simpson’s analysis were applied to evaluate the alpha diversity of OTUs, and the results indicate that the OTUs exhibited a significant difference between the WT and DOM groups (Figure 4A, *P* < 0.05). Meanwhile, hierarchical clustering, PCA, and PLS-DA analyses were also conducted to evaluate the impact of DOM on the intestinal microbiome composition. Results indicate the microbes in the WT and DOM groups were clustered into different subsets at the phylum level due to the microbial diversity (Figures 4B–D). Additionally, microbes of Proteobacteria, Firmicutes, and Actinobacteriota show the most abundant diversity at the phylum level in both the WT and DOM groups. Notably, the proportion of the members of the phylum Proteobacteria was significantly increased from 74.48% (WT) to 97.22% (DOM) (*P* = 0.0007, Figure 4B).

### Microbial Comparison and Interaction Analysis at Phylum Level in *A. grunniens* Intestine Domesticated With Compound Feed

**FIGURE 5:**
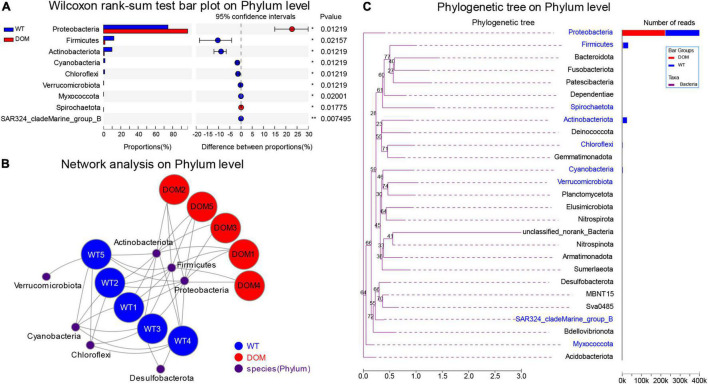
Microbial comparison and interaction analysis at phylum level in *A. grunniens* intestine domesticated with compound feed. **(A)** Differentially colonized microbial communities at phylum level; **(B)** interaction network between microbial communities and *A. grunniens* individuals; and **(C)** Phylogenetic analysis at the phylum level.

Next, the microbial composition and interaction analysis was conducted to reveal the dominant microbiota in both WT and DOM groups. A total of nine phyla were identified as the dominant microbes in the intestine of the DOM group, including upregulated Proteobacteria and Spirochaetota, and downregulated Firmicutes, Actinobacteriota, Cyanobacteria, Chloroflexi, Verrucomicrobiota, Myxococcota, and SAR324_cladeMarine_group_B (*P* < 0.05, Figure 5A). The interactions between microbes and samples are shown in Figure 5B, which indicates the members of phyla Proteobacteria, Firmicutes, and Actinobacteriota were dominant in both the WT and DOM groups, while Cyanobacteria, Chloroflexi, and Verrucomicrobiota were mainly dominant in the WT groups. Additionally, the phylogenetic analysis of these dominant microbes was also conducted, which revealed Proteobacteria exhibited a far distance from other microbes at the phylum level in evolution (Figure 5C).

### Microbial Comparison and Interaction Analysis at Genus Level in *A. grunniens* Intestine Domesticated With Compound Feed

**FIGURE 6:**
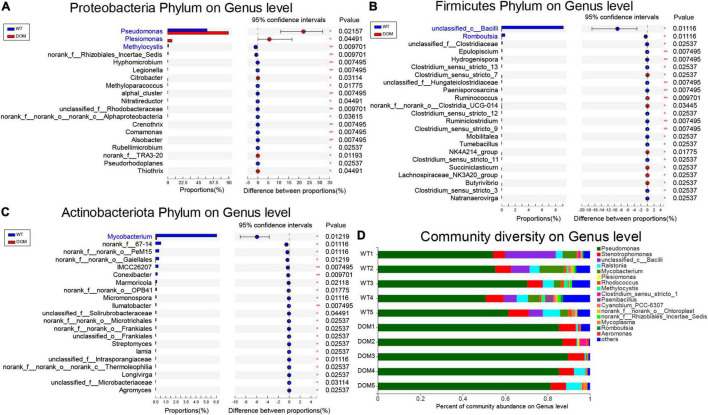
Microbial comparison and interaction analysis at genus level in *A. grunniens* intestine domesticated with compound feed. **(A–C)** Differentially colonized microbial communities at genus level of **(A)** phylum Proteobacteria; **(B)** phylum Firmicutes; and **(C)** phylum Actinobacteriota. **(D)** Community diversity at the genus level.

To further explore the underlying microbial regulation on feed domestication, the alternation at the genus level was analyzed. The microbes of the phylum Proteobacteria, *Pseudomonas*, *Plesiomonas*, and *Methylocystis* were the most abundant and dominant microbes in the DOM group (Figure 6A). Meanwhile, *unclassified_c_Bacilli* and *Romboutsia* were the most abundant and dominant microbes in the DOM group for the phylum Firmicutes (Figure 6B), and *Mycobacterium* was the most abundant and dominant microbe in the DOM group for the phylum Actinobacteriota (Figure 6C). Additionally, analysis of the community diversity revealed that the genus *Pseudomonas* was the most dominant and diverse microbe under feed domestication, which increased from 58.47% in the WT group to 89.77% in the DOM group (Figure 6D).

### Integrated Analysis Between Differentially Expressed Genes and Microbes Under Feed Domestication in *A. grunniens*

**FIGURE 7:**
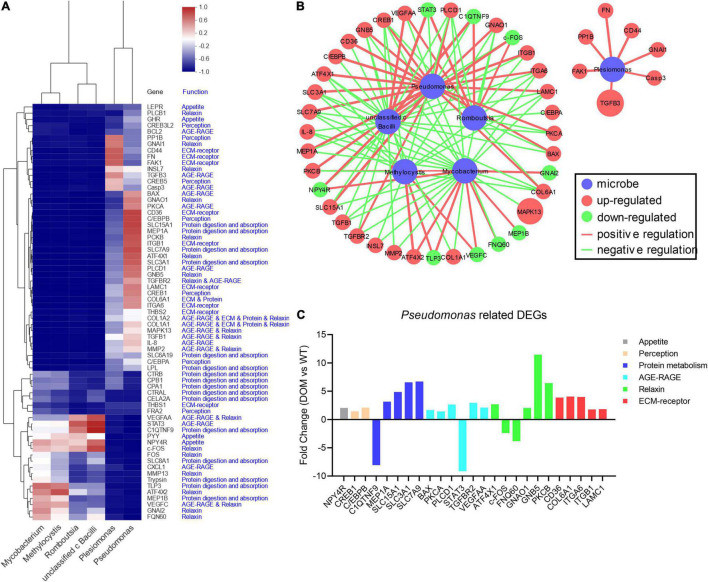
Integrated analysis between DEGs and microbes under feed domestication in *A. grunniens*. **(A)** Heatmap cluster between key microbes at genus level and enriched genes. **(B)** Interacting regulatory network between key microbes and key enriched genes. In the network, nodes in red color represent the upregulated DEGs, in green color represent the downregulated DEGs, in blue color represent the microbes. The size of a node represents the mRNA abundance retrieved from RNA-seq, and the width of the connecting line represents the correlation intensity between DEGs and microbes. **(C)** Transcriptome profiles of *Pseudomonas*-mediated DEGs relate to appetite, perception, protein digestion and absorption (protein metabolism), AGE-RAGE, relaxin, and ECM–receptor signaling.

The integrated network analysis between DEGs and microbes was performed to uncover the underlying mechanism of feed domestication. With Pearson analysis, a total of 67 DEGs co-related with six dominant microbes were applied to the heatmap cluster. Results revealed that the microbes were clustered into three sub-clusters based on their correlations with DEGs: *Pseudomonas* and *Plesiomonas* that belong to the phylum Proteobacteria were clustered into the same sub-cluster, *Romboutsia* and *unclassified c Bacilli* that belong to the phylum Firmicutes were clustered into the same sub-cluster, while *Methylocystis* in the phylum Proteobacteria and *Mycobacterium* in the phylum Actinbacteriota were clustered into another sub-cluster (Figure 7A). Meanwhile, DEGs (*P*-value < 0.05 in correlation with key microbes) from the heatmap cluster were enriched in the integrated network analysis, shown in Figure 7B. From the results, 44 DEGs were found to functionally interact with the microbes, including 25 DEGs that were closely related to *Pseudomonas*. Unexpectedly, the microbe *Plesiomonas*, which belongs to the phylum Proteobacteria along with *Pseudomonas*, did not directly interact with *Pseudomonas* in the network, revealing their different regulatory mechanisms under domestication. Additionally, the expression of *Pseudomonas-*related DEGs retrieved form RNA-seq was also analyzed, which indicated the regulatory role of *Pseudomonas* in appetite, perception, protein digestion and absorption, AGE-RAGE, relaxin, and ECM–receptor signaling pathways (Figure 7C).

### Protein Digestion and Absorption, and AGE-RACE-Mediated Apoptosis Were Active to Feed Domestication in *A. grunniens*

**FIGURE 8:**
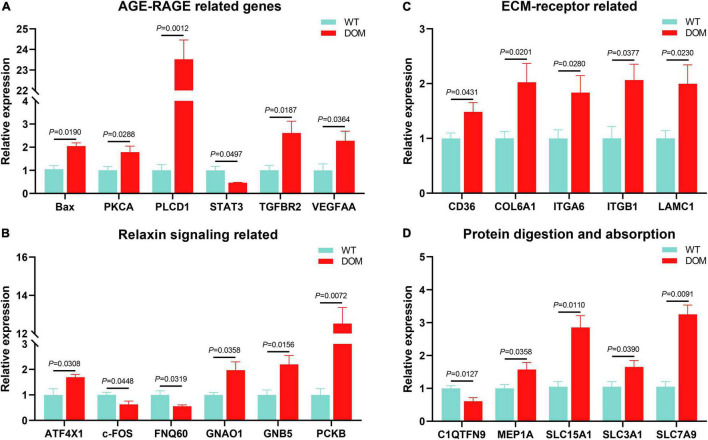
*Pseudomonas* medicated AGE-RAGE, Relaxin, ECM-receptor, and Protein digestion and absorption were active to feed domestication in *A. grunniens*. Transcriptional expression of AGE-RAGE related **(A)**, Relaxin signaling related **(B)**, ECM-receptor related **(C)**, and protein digestion and absorption related **(D)** genes. Data were analyzed by Students’ *t*-test, results were indicated as mean ± SEM, *n* = 9.

The above-mentioned results indicate that cellular homeostasis (featured as AGE-RACE, relaxin, and ECM-receptor signaling) and protein digestion and absorption were involved in feed domestication. Next, we evaluated the expression of the key genes in these signaling pathways. The genes related to AGE-RAGE signaling (Figure 8A), such as *Bax* (*P* = 0.0190), *PKCA* (*P* = 0.0288), *PLCD1* (*P* = 0.0012), *TGFBR2* (*P* = 0.0187), and *VEGFAA* (*P* = 0.0364) were up-regulated in DOM, while *STAT3* (*P* = 0.0497) was down-regulated in DOM. Meanwhile, genes related to relaxin signaling (Figure 8B), such as *ATF4X1* (*P* = 0.0308), *GNAO1* (*P* = 0.0358), *GNB5* (*P* = 0.0156), and *PKCB* (*P* = 0.0072) were up-regulated, while *c-FOS* (*P* = 0.0448) and *FQN60* (*P* = 0.0319) were down-regulated in DOM. Accordingly, genes related to ECM-receptor signaling (Figure 8C), such as *CD36* (*P* = 0.0431), *COL6A1* (*P* = 0.0201), *ITGA6* (*P* = 0.0280), *ITGB1* (*P* = 0.0377), and *LAMC1* (*P* = 0.0230) were all up-regulated in DOM. Additionally, genes related to protein digestion and absorption (Figure 8D), such as *C1QTNF9* (*P* = 0.0127), *MEP1A* (*P* = 0.0358), *SCL15A1* (*P* = 0.0110), *SLC3A1* (*P* = 0.0390), and *SLC7A9* (*P* = 0.0091) were all up-regulated in DOM. These data reveal that *Pseudomonas* activates protein utilization and cellular homeostasis in response to feed domestication.

”

### Hypothetical Schematic of *Pseudomonas-*Mediated Ingestion and Growth Performance Regulated by Feed Domestication in *A. grunniens*

**FIGURE 9:**
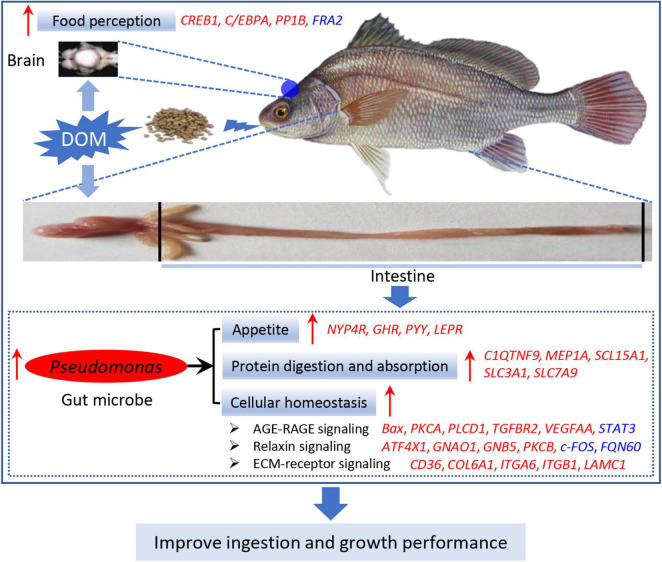
Hypothetical schematic of *Pseudomonas-*mediated ingestion and growth performance regulated by feed domestication in *A. grunniens*. Hypothetical regulation of *Pseudomonas* on ingestion and growth performance was raised based on the results in this study. Feed domestication improved feed perception in the brain tissue, thereby transducing the signal to the intestine. In the intestine, DOM improved the abundance of microbiota of *Pseudomonas*, eventually improving the ingestion and growth performance by enhancing appetite, protein digestion and absorption, and cellular homeostasis capacity. Arrows in red color represent the positive regulation or activation, italic letters in red color represent the upregulated genes, and italic letters in blue color represent the downregulated genes.

Based on the above-mentioned results, we raised the hypothetical regulation schematic of microbiota *Pseudomonas* (Figure 9). Feed domestication improved the food perception in the brain tissue, thereby transducing the signal to the intestine. In the intestine, DOM enhanced the abundance of the microbiota of *Pseudomonas*, which specifically plays a vital role in the activation of the appetite, protein digestion and absorption, and cellular homeostasis capacity. Therefore, *Pseudomonas* is the dominant microbiota, and its functions are important to improve the ingestion and growth performance during feed domestication.

## Discussion

Aquaculture is the fastest growing food-producing sector worldwide (FAO, 2020). The continued success of this sector relies on the productivity of fish culture by overcoming common challenges, such as the availability of excellent germplasm resources, advanced breeding management, and reasonable nutritional and environmental management programs (FAO, 2020). However, despite the fact that the cultivation practice has been well developed, over 90% of the production involves just 27 species (Hallerman et al., 2021). Therefore, the limited availability of well-developed species might become another bottleneck for aquaculture development in the next few decades. Herein, the domestication of aquatic species with high prolificacy, disease resistance, and rapid growth rate is of great importance (Teletchea and Fontaine, 2014; Xiong et al., 2014; Chen et al., 2017). Accumulating evidence has shown that inherited differences are closely associated with the feeding habits in the aquatic animals, as demonstrated in tilapia (Xiong et al., 2014), sticklebacks (Purnell et al., 2007), Eurasian perches (Chen et al., 2017), and mandarin fish (Shen et al., 2021). This inspired us to develop adaptive traits from the natural precious species to meet the high-quality protein requirements of humans.

One of the mechanisms influencing the feeding behavioral traits could be the changes in the host gut microbiome. Evidence suggests that microbial diversity is a marker of a healthy intestinal microbiome (Lozupone et al., 2012). Generally, microbiome diversity is relatively lower in diseased individuals compared to that of healthy individuals in humans (Cortes et al., 2022) and mice (Zhang et al., 2021). However, studies also reveal that starvation could increase the intestinal microbial diversity in desert locusts (Dillon et al., 2010) and fish (Xia et al., 2014). In addition, individuals with pure diets had less diverse microbiota than the fish feeding with mixed diets (Bolnick et al., 2014). These contrasting results indicate gut microbiota exerts different colonization resistance patterns under different enteric threats. In our present study, DOM decreased the microbial diversity in compare with WT, which might be a multiple effects of ingestion behavior, dietary intake, and intestinal microbiome rebalance.

In addition to microbial diversity, the relative abundance of microbes directly reveals the function of specific gut microbes. Generally, all species have species-specific gut microbiome composition, which influences their adaptation and diversification behaviors, including food preference, phenotypic plasticity, and innate and adaptive immunity (Glazko et al., 2021). The intestinal microbiota is either indigenous or transient. The indigenous genera are mainly obtained from the parents (Thomas and Percival, 2009), while the transient ones are largely dependent on the diet (Ringø et al., 2016). Fish with different dietary habits have distinct transient gut microbial communities: carnivorous fish are typically abundant in the phylum Proteobacteria, which functions importantly in the digestion and absorption of proteins (Ray et al., 2012). Meanwhile, herbivorous fish are enriched with Clostridiales, Bacteroidales, and Verrucomicorbiales; omnivorous fish had increased populations of Rhizobiales, Fusobacteriales, and Planctomycetales; while both omnivorous and carnivorous fish have abundant populations of Desulfovibrionales and Aeromonadales (Sullam et al., 2012; Liu et al., 2016). Additionally, dietary intake could affect the structure of the fish gut microbiome. In general, plant-derived dietary proteins have been linked to significantly reduced diversity of microbiota (Desai et al., 2012), with an increase in the relative abundance of Lactobacillales, Bacillales, and Pseudomonadales (Michl et al., 2017), while animal-derived proteins nurture Bacteroidales, Clostridiales, Vibrionales, Fusobacteriales, and Alteromonadales in the gut (Michl et al., 2017). In the present study, phylum Proteobacteria was significantly increased in the gut of freshwater drum, indicating that the protein digestion and absorption capacity were enhanced under feed domestication, which was in accordance with the results of digestive enzyme activity in the present study. Consistently, the abundance of the phylum Verrucomicrobiota, which is commonly found in herbivorous fish, was decreased. In addition, our data are in line with the previous studies that reported Proteobacteria, Firmicutes, and Actinobacteria (in this order of abundance) as the most common phyla of intestinal bacteria in fish (Butt and Volkoff, 2019; Silva-Brito et al., 2021).

The phylum Proteobacteria has the largest phylogenetic composition, comprising 116 validated bacterial families (Shin et al., 2015). Meanwhile, the population of Proteobacteria is the most unstable among the four main phyla (Firmicutes, Bacteroidetes, Proteobacteria, and Actinobacteria) in the gut microbiota over time (Faith et al., 2013). The expansion of Proteobacteria is sensitive to environmental factors, such as diet (Caporaso et al., 2011). In humans, the abundance of Proteobacteria in the gut is low under healthy conditions, while an abnormal expansion of Proteobacteria is associated with inflammation or disease conditions, such as gastric bypass (Liou et al., 2013), metabolic disorders (Fei and Zhao, 2013), and inflammation and cancer (Morgan et al., 2012). However, the exact function of Proteobacteria in the fish remains unclear. Biologically, Proteobacteria are the major source to induce gene dysregulation in animals (Bradley and Pollard, 2017). To assess which taxa contributed to the variable expression of genes, we first conducted interaction and community diversity analyses at the genus level. The results indicate that the most abundant genus *Pseudomonas* was increased and occupies the most proportion of alternation.

Over the past decades, the crosstalk between the gut microbiota and the brain has received dramatic attention (Zheng et al., 2021). The microbiota–gut–brain axis is a bidirectional signaling pathway mediating the crosstalk between the microbiota, the intestine, and the central nervous system (CNS) (Cryan et al., 2019). CNS regulates the activities of the intestine and the microbiota, while the signals originating from the microbiota and the intestine affect the development and the function of the CNS inversely (Lynch and Pedersen, 2016). A wide array of signaling molecules that convey information about whole-body nutritional status have been identified and studied, including neuropeptides, hormones, nutrients, and metabolites produced by peripheral and CNS tissues in response to changes in the nutrition and environment (Richards and Proszkowiec-Weglarz, 2007). In the present study, the levels of metabolic enzymes like amylase, lipase, and trypsin; neuropeptides of NPY4R and PYY; hormones of GHR; functional proteins of LEPR, CREB1, FRA2, C/EBPA, and PP1B were all increased in the DOM group, indicating the appetite and perception were activated. Meanwhile, *Pseudomonas* was confirmed to regulate appetite-related NPY4R and perception-related CREB1 and C/EBPB in the study, indicating the crosstalk between microbiome *Pseudomonas* and CNS. As the appetite and perception are regulated by the central nervous system (Isganaitis and Lustig, 2005), the gut microbiota *Pseudomonas* might improve the ingestion performance of freshwater drums by crosstalk with the central nervous system.

Additionally, *Pseudomonas* was identified to have the genes involved in protein metabolism and cellular homeostasis signaling (AGE-RAGE, relaxin, and ECM-receptor signaling), which was in accordance with the results of RNA-seq analysis. However, there are limited reports demonstrating the regulation between gut *Pseudomonas* and the host protein metabolism and cellular homeostasis. In addition, reports indicate metabolic homeostasis is associated with appetite regulation (Sloth et al., 2007; Karl et al., 2016; Hopkins and Blundell, 2017). Therefore, we hypothesize that *Pseudomonas* impacts protein metabolism and cellular homeostasis by the mediator of CNS. These data reveal the ingestion behavior of freshwater drums is closely related to the central nervous system, the gut, and the gut microbiota *Pseudomonas*. Based on these results, future efforts in the development of feed demonstration strategies for a freshwater drum by targeting *Pseudomonas* and its medicated genes are warranted.

## Conclusion

In this study, feed domestication improves ingestion, digestion, and growth performance, enhances protein digestion and absorption, and cellular homeostasis (Relaxin, ECM-receptor, and AGE-RAGE signaling). *Pseudomonas* was the most abundant microbe interacting with DEGs in response to feed domestication. These results indicate the *Pseudomonas-*mediated protein utilization and cellular homeostasis play a prominent role in ingestion performance during feed domestication. Consequently, *Pseudomonas* and related DEGs could be the target to improve the food performance and domestication in freshwater drum *A. grunniens*.

## Data Availability Statement

The datasets presented in this study can be found in online repositories. The names of the repository/repositories and accession number(s) can be found below: NCBI BioProject, PRJNA801055.

## Ethics Statement

The animal study was reviewed and approved by the Animal Care and Use Committee of Nanjing Agricultural University (Nanjing, China).

## Author Contributions

CS: conceptualization, methodology, investigation, software, writing–original draft, and writing–review and editing. HW: data curation and resources. GaL: investigation and validation. XM, GoL, and MX: investigation and editing. NW: investigation and visualization. JC: investigation and software. HL: supervision and writing–original draft. PX: funding acquisition and supervision. All authors contributed to the article and approved the submitted version.

## Conflict of Interest

The authors declare that the research was conducted in the absence of any commercial or financial relationships that could be construed as a potential conflict of interest.

## Publisher’s Note

All claims expressed in this article are solely those of the authors and do not necessarily represent those of their affiliated organizations, or those of the publisher, the editors and the reviewers. Any product that may be evaluated in this article, or claim that may be made by its manufacturer, is not guaranteed or endorsed by the publisher.

## Supplementary Material

Supplementary Table 1 Differentially expressed genes (DEGs) under feed domestication in *A. grunniens* intestine. **(A)** Upregulated DEGs; **(B)** downregulated DEGs. Transcripts with | fold change| ≥ 2 and corrected *P*-value < 0.05 were identified as DEGs.

Supplementary Table 2 GO and KEGG enrichment of DEGs under feed domestication in *A. grunniens* intestine. **(A)** GO items of DEGs; **(B)** KEGG items of DEGs.

Supplementary Table 3 Operational taxonomic units (OTUs) of the microbiota identified from the intestine of *A. grunniens* under feed domestication. OTUs were identified and annotated at the level of phylum, class, order, family, genus, and species, respectively.

Supplementary Table 4 Pearson analysis between DEGs and microbes.
