# Corrigendum: Gut microbes reveal *Pseudomonas* medicates ingestion preference via protein utilization and cellular homeostasis under feed domestication in freshwater drum, *Aplodinotus grunniens*

**DOI:** 10.3389/fmicb.2023.1259988

**Published:** 2023-08-24

**Authors:** Changyou Song, Haibo Wen, Guangxiang Liu, Xueyan Ma, Guohua Lv, Ningyuan Wu, Jianxiang Chen, Miaomiao Xue, Hongxia Li, Pao Xu

**Affiliations:** ^1^Key Laboratory of Freshwater Fisheries and Germplasm Resources Utilization, Ministry of Agriculture and Rural Affairs, Freshwater Fisheries Research Center, Chinese Academy of Fishery Sciences, Wuxi, China; ^2^Wuxi Fisheries College, Nanjing Agricultural University, Wuxi, China

**Keywords:** feed domestication, microbe, *Pseudomonas*, protein utilization, *Aplodinotus grunniens*

In the published article, there was an error in the Funding statement. The grant number for funding from the Innovation Project of Jiangsu Agricultural Science and Technology was incorrectly presented as “SCX(20)2268”. The correct Funding statement appears below.

